# Supplementary material for: Motor crosslinking augments elasticity in active nematics
Source: ArXiv. 2023 Aug 31:arXiv:2308.16831v1. Preprint. [Version 1] (PMC10491317)
Supplement: 1 [file NIHPP2308.16831V1-supplement-1.pdf]

SUPPLEMENTAL FIGURES

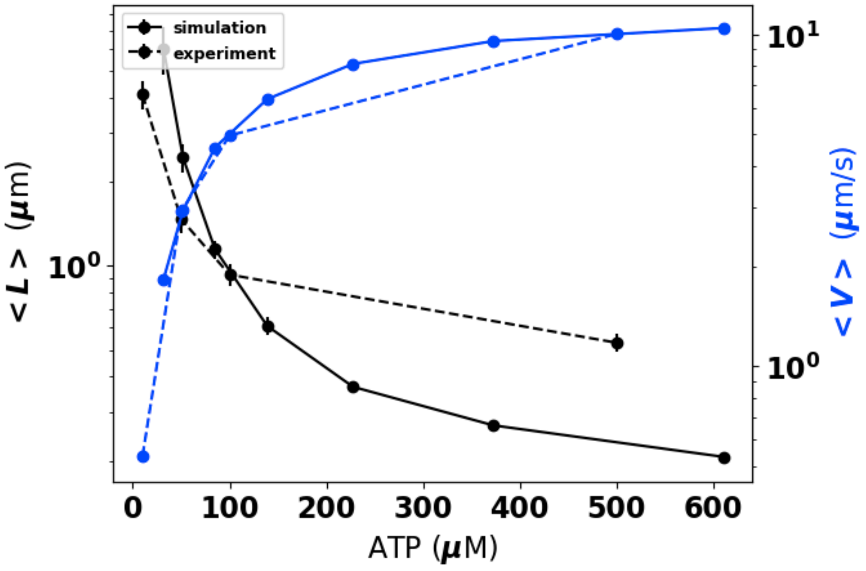

Figure S1: Simulations reproduce single filament velocity and run length trends. Single filament motor velocity (blue) and single filament run length (black) from experiments (dashed lines) and simulaitons (solid lines) over a range of ATP concentrations. The final rates we compute after 10,000 tuning steps are 1821 s^{-1}, 932 s^{-1}, 6 $s^{-1}\mu$M ATP.

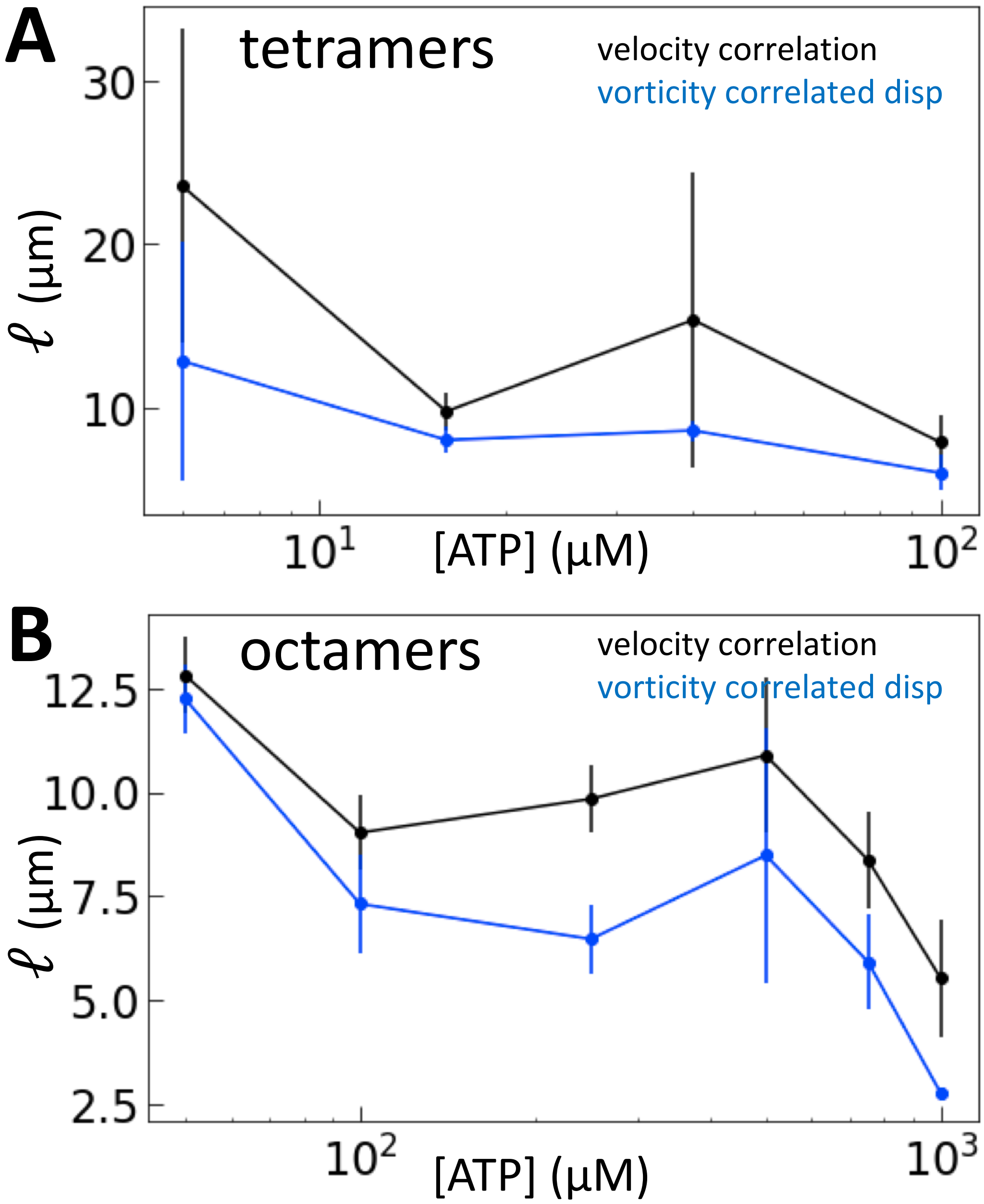

Figure S2: ℓ_{vort} **robustly captures nematic length scale.** Comparison of ℓ_{vort} as calculated in [35] and the traditional velocity correlation length — $C_{vv} = 1/e$ — for nematics driven by 120 pM tetramers (A) and 50 pM octamers (B). Errorbars are averages over five separate frames for C_{vv} and five 10s snippets for ℓ_{vort}.

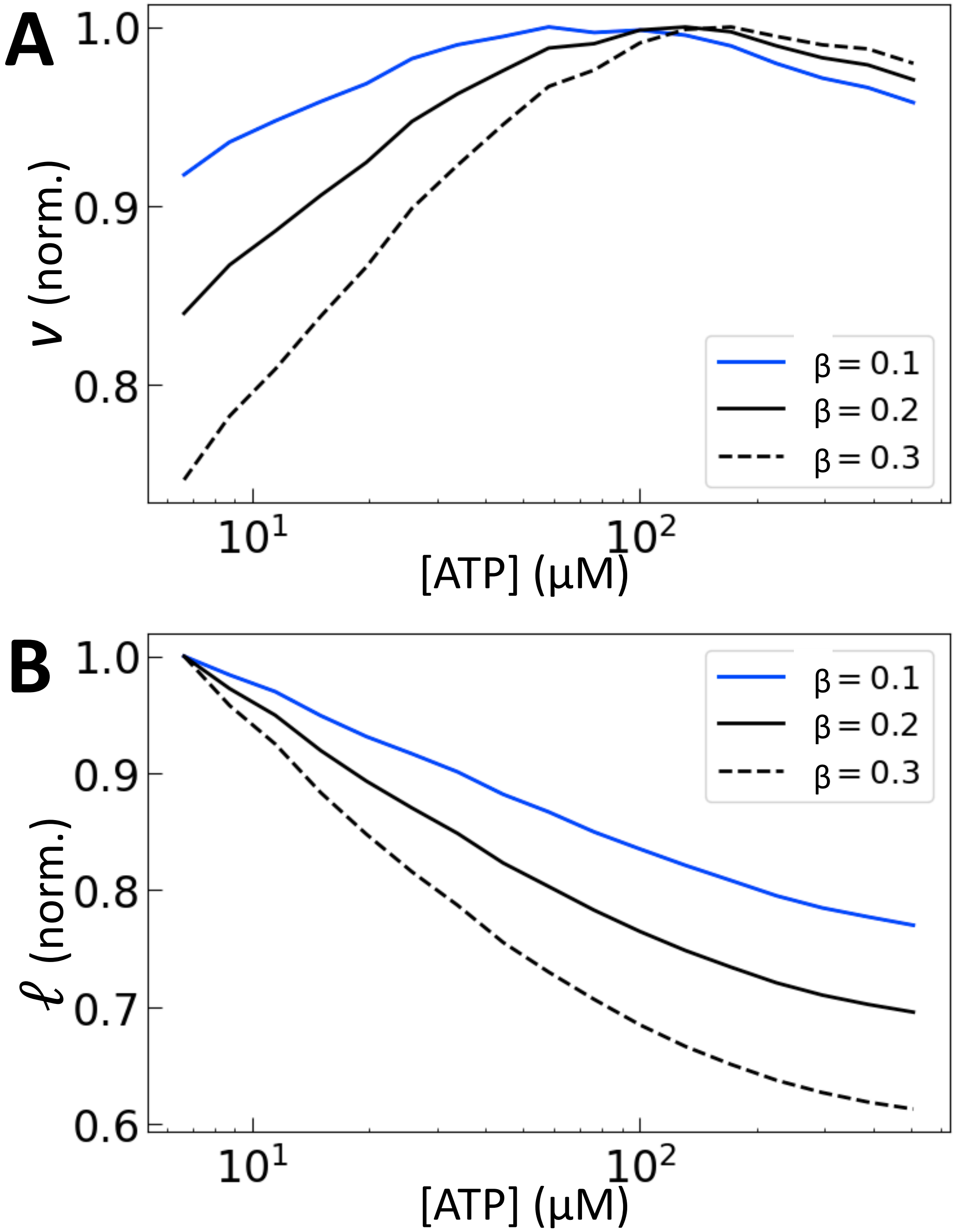

Figure S3: Strong activity coupling decreases nonmonotonicity in tetrameric driven nematics. v (A) and ℓ (B) from scaling predictions for different coupling exponents β.

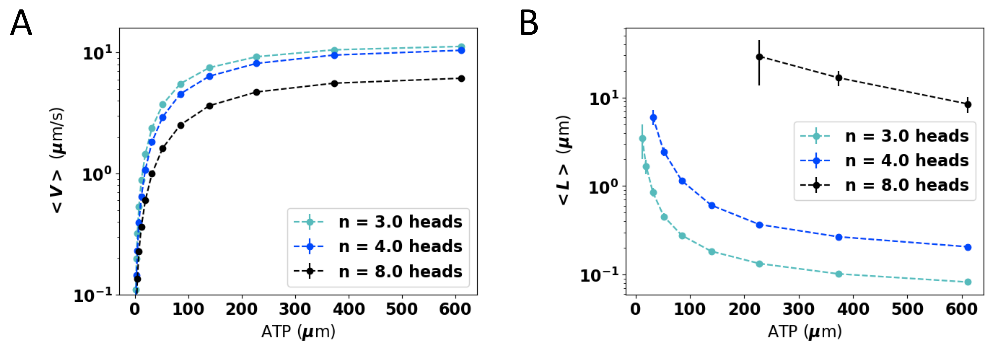

Figure S4: Motor velocity and run length are cluster valency dependent phenomena. Single filament motor velocity (A) and single filament run length (B) measured from simulation for clusters with 3,4, or 8 heads per cluster.

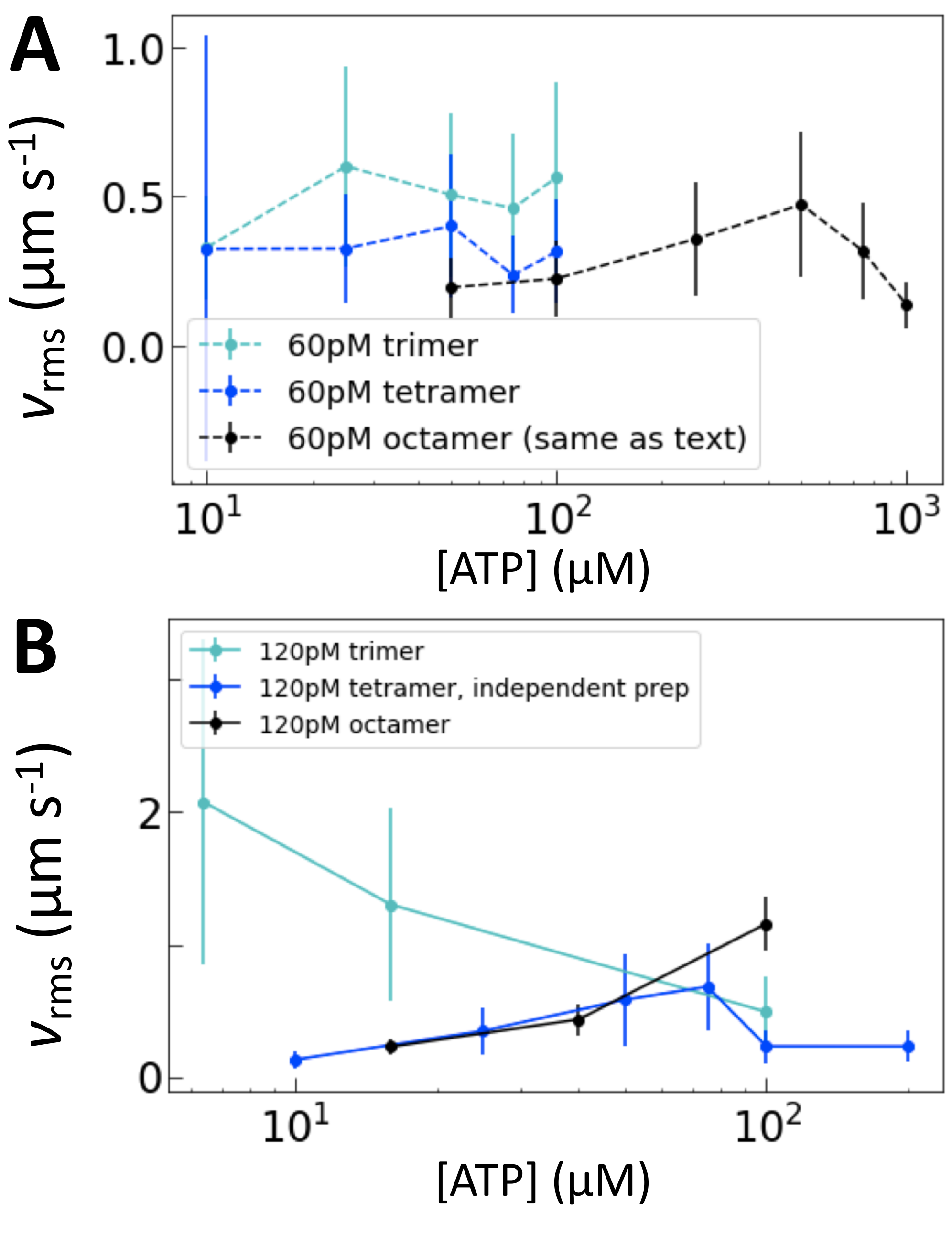

Figure S5: Peak shift is robust across days and motor concentrations. v_{rms} for independent replicates of oligomerization data at 60 pM (A) or 120 pM motor clusters. All data are different from those in the text except for the octamers in (A) which are included as a reference. Error bars are standard deviations of v_{rms} over 100 s of steady-state activity.

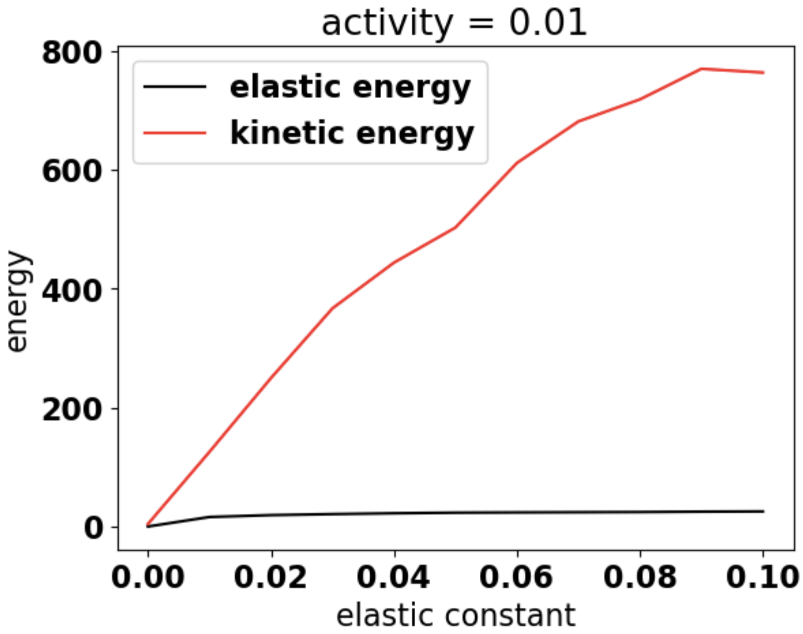

Figure S6: Nematic elasticity increases energy in the nematic. Elastic (black) and kinetic (red) energy for nematics in lattice Boltzmann simulations with constant $\alpha = 0.01$ across a range of K.
